# Data Collection Based on Opportunistic Node Connections in Wireless Sensor Networks

**DOI:** 10.3390/s18113697

**Published:** 2018-10-30

**Authors:** Guisong Yang, Zhiwei Peng, Xingyu He

**Affiliations:** 1 Department of Computer Science and Engineering, School of Optical-Electrical and Computer Engineering, University of Shanghai for Science and Technology, Shanghai 200093, China; gsyang@usst.edu.cn (G.Y.); zhiwei-peng@outlook.com (Z.P.); 2 Shanghai Key Lab of Modern Optical Systems, Shanghai 20093, China; 3 Public Experiment Center, University of Shanghai for Science and Technology, Shanghai 200093, China

**Keywords:** working–sleeping cycle strategy, wireless sensor networks, opportunistic node connections, random graph

## Abstract

The working–sleeping cycle strategy used for sensor nodes with limited power supply in wireless sensor networks can effectively save their energy, but also causes opportunistic node connections due to the intermittent communication mode, which can affect the reliability of data transmission. To address this problem, a data collection scheme based on opportunistic node connections is proposed to achieve efficient data collection in a network with a mobile sink. In this scheme, the mobile sink first broadcasts a tag message to start a data collection period, and all nodes that receive this message will use the probe message to forward their own source information to the mobile sink. On receiving these probe messages, the mobile sink then constructs an opportunistic connection random graph by analyzing the source information included in them, and calculates the optimal path from itself to each node in this random graph, therefore a spanning tree could be generated with the mobile sink play as the root node, finally, it broadcasts this spanning tree so that each node could obtain an optimal path from itself to the mobile sink to forward the sensing data. In addition, a routing protocol that adapts to different nodes operating statuses is proposed to improve the reliability of data transmission. Simulation results show that the proposed scheme works better concerning the packet delivery rate, energy consumption and network lifetime.

## 1. Introduction

As a popular way of information access, Wireless Sensor Networks (WSNs) have been widely used in smart industrial fields [1] to undertake tasks such as factory automation, fault diagnosis, fuel consumption monitoring, surveillance, and industrial control, etc. Their application usually involves a large number of static sensor nodes (e.g., photoelectric sensors, ultrasonic sensors, gas sensors, video sensors, etc.) deployed over vast areas to perform data sensing, gathering and communication. However, the sensor nodes in WSNs have limited power supply that is not easy to replenish, which restricts their long-term and sustained work.

The use of mobile sinks in WSNs to replace the traditional static base stations for data collection is believed to be able to improve the network lifetime, and the feasibility of this scheme is demonstrated in details in Reference [2]. Usually, the mobile sink has stronger computing power than common sensor nodes and its energy can be replenished in a timely way. Depending on the particular application needs, the mobile sink can be either part of the external environment or part of the network, and its movement trajectory can also be either controlled or randomly planned [3]. By properly designing the movement trajectory for the mobile sink, the hot-spot problem [4] in traditional networks can be avoided and the energy consumption of sensor nodes can be more evenly balanced, thus prolonging the network lifetime. To fully explore the role of mobile nodes, in Reference [5], the authors proposed a joint energy replenishment and data collection algorithm, in which a mobile charger is used to replenish the energy for nodes and it can function as a mobile sink when equipped with wireless transceivers. In Reference [6], the authors proposed a Wi-Fi-based passive cyber–physical social sensing approach for tracking people’s behaviors by sensing Wi-Fi messages from mobile devices people carry with them. However, these studies did not further consider changing the node operating status to achieve a better network lifetime performance.

To cope with this challenge, researchers have proposed the working–sleeping cycle strategy for sensor nodes, in which nodes can go to sleep to save energy while they are free of work. These studies usually focus on designing node-scheduling schemes to accomplish data transmission, in which the working–sleeping cycle strategy can be categorized into synchronous and asynchronous [7]. Therefore, the changeable node operating status in this strategy is an advantage of these studies, which can prolong the network lifetime. However, the working–sleeping cycle strategy for nodes may cause opportunistic node connections, which results in link instability and affects the data collection efficiency.

In fact, the reasons why opportunistic node connections exist in WSNs can be summarized as follows: (1) WSNs are often deployed in harsh environments where wireless signals are susceptible to interference, which may cause link instability [8], further leading to opportunistic node connections; (2) the movement of nodes usually leads to intermittent connected links and creates some form of opportunistic node connections [9]; (3) due to the limited energy of the nodes, in some scenarios the sensor nodes adopt a working–sleeping cycle strategy to save energy, and accordingly, the adjacent nodes may not be able to communicate with each other continuously [10], thus bringing about opportunistic node connections.

**Figure 1:**
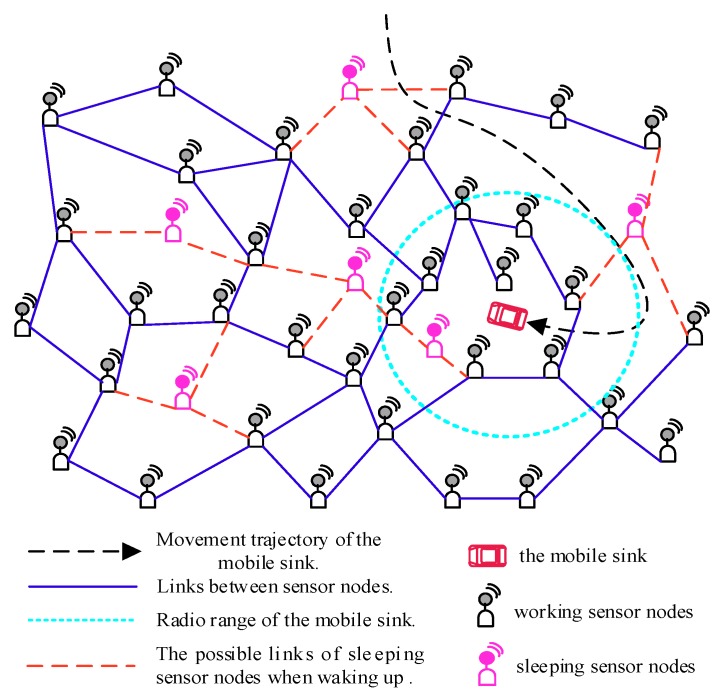
Illustration of a Wireless Sensor Network (WSN) with opportunistic node connections, in which sensor nodes adopt an asynchronous working–sleeping cycle strategy to save energy.

This work considers that all sensor nodes adopt an asynchronous working–sleeping cycle strategy to save energy and a mobile sink is used to collect data in the network, as shown in Figure 1, where the network works based on opportunistic node connections since they may not be able to communicate with each other continuously. In this case, how to model this kind of WSNs to design a scheme, and based on which to ensure the reliability of data transmission in the network becomes a meaningful challenge.

To deal with this problem, the random graph theory originated by the pioneering work of Erdős and Rényi [11] was adopted to model WSNs, in which all sensor nodes are represented by graph vertices while all wireless links are represented by edges. All edges have a probability to reflect the quality of wireless links. The random graph theory that is used in modeling WSNs can be called a random geometric graph, in which an edge can only exist between two adjacent nodes [12]. To make the model more appropriate to the real situation of WSNs, Ren et al. [13] took the reliability of links into consideration to present a novel random geometric graph coverage model. Moreover, Dong et al. [14] described a range-dependent model for WSNs by using random graph theory, of which the probability of an edge existing was expressed as a function of the distance between its two end vertices in the fading environment.

In this work, by taking the randomness of opportunistic node connections into account, we propose a novel random graph model named Opportunistic Connection Random Graph (OCRG) for WSNs. Inspired by the work of Fu et al. [15], who first proposed a scheme to address the connectivity between a designated source and destination in random graphs, in which they assumed that at each step one edge could be tested to see if it existed or not and an optimal policy that minimized the total expected number of steps determined while establishing whether the designated source and destination were connected. As a matter of fact, this work mainly focuses on the connectivity between any source node and the mobile sink in the OCRG, and based on the opportunistic node connections, a data collection scheme is proposed, in this scheme, all nodes can use the calculated optimal path to forward their sensing data to the mobile sink.

In the scheme, first, the mobile sink can launch a data collection task anywhere in network at any time by broadcasting a tag message that declares its data collection period, and each node that receives this message will forward its source information (including its whole working time in this data collection period, its status transition frequency in this period and all its neighbors’ IDs) included in a probe message to the mobile sink. Here the energy efficient probe message forwarding mechanism is designed to minimize the overall energy consumption. On receiving these probe messages, the mobile sink first constructs an opportunistic connection random graph by analyzing the source information included in them, then calculates the optimal path from itself to each node in this random graph, and hence a spanning tree could be generated where the mobile sink play as the root node, and finally it broadcasts this spanning tree so that each node can obtain the optimal path from itself to the mobile sink, and according to which to forward the sensing data. In addition, a routing protocol which adapts to different nodes operating statuses is proposed to improve the reliability of data transmission when nodes forwarding their sensing data along the calculated optimal path. The experimental results show that this scheme can effectively improve the reliability of the data transmission while prolonging the network lifetime. The main contributions of this paper are summarized as follows:

1. An opportunistic connection random graph is constructed to reflect the opportunistic node connections caused by an asynchronous working–sleeping cycle strategy;
2. For each node, an energy efficient probe message forwarding mechanism is used to minimize the overall energy consumption in network when forwarding the source information to the mobile sink;
3. A routing protocol which adapts to different nodes operating statuses is proposed to improve the reliability of data transmission when nodes are forwarding their sensing data along the calculated optimal path.

The rest of the paper is organized as follows: Section 2 describes the related work. Section 3 gives the system model. Section 4 presents the proposed data collection scheme in details. Simulation results and performance evaluations are shown in Section 5. Finally, Section 7 summarizes this work and gives some future research directions.

## 2. Related Work

In traditional WSNs, all sensor nodes including the sink nodes are static, sensor nodes near the sink have more traffic loads for they have to participate in forwarding sensing data from all other nodes to the sink, therefore they easily exhaust their limited energy, and which will cause network partition. This problem is called the hot-spot problem [16] and it has an adverse effect on the network lifetime.

To address this problem, in recent years researchers have introduced the mobile sink concept to networks. By fully exploiting the mobility of the mobile sink, the nodes around the sink could change continually, therefore the energy consumption in routing is distributed more evenly in the network, and thereby it prolongs the network lifetime. According to the work of the previous researchers, the path design for mobile sinks can be classified into predefined paths [17], controlled paths [18], or uncontrolled (random) paths [19]. By employing these paths, the mobile sink can play an important role in scenarios where nodes suffer opportunistic connections. For example, in Reference [20], the authors considered that the WSNs deployed within hostile environments might suffer from large-scale damage where many sensors failed simultaneously and this would cause the WSNs to split into disconnected segments, and they proposed a two-step heuristic for connecting isolated segments through intermittent links, in which a set of mobile nodes served as mobile data carriers. In Reference [21], the authors considered that in mobile sensor networks, the connectivity between nodes was intermittent due to the blockage of radio signals and nodal mobility, and they proposed a reliable data delivery scheme for the networks with an enhanced delay technique, in which nodes estimated the connectivity and expected inter-encounter time with sink nodes.

All these approaches can equalize the energy consumption of nodes, but they are not flexible enough to prolong the network lifetime because they have not considered designing different node operating statuses. Therefore, the working–sleeping cycle strategy was introduced to WSNs, in which researchers considered that the proper scheduling of sensor nodes to sleep or work could effectively reduce their unnecessary energy consumption, thus prolonging the network lifetime.

According to the node operating status, the researches can easily be divided into two main categories. In the first category, the studies aimed at synchronous working–sleeping cycle strategy, for example, in Reference [22], the authors proposed an event-triggered sleeping energy mode for the synchronous working–sleeping cycle strategy of sensor nodes, in which nodes with no data packets immediately entered sleeping status after the synchronization phase and did not participate in media access contention. However, the synchronization process remains a significant contributor to the energy consumption. To solve the problem, Ng et al. [23] proposed an energy-efficient synchronization algorithm that could reduce energy consumption by adaptively regulating the synchronization traffic and synchronization wakeup period based on the changing network neighborhood conditions through counter-based and exponential-smoothing algorithms. In the second category, the studies focus on asynchronous working–sleeping cycle strategy, in which each node could have an independent working–sleeping scheduling, by scheduling a certain number of nodes to work; they can achieve the coverage for a specific area and ensure the network connectivity in it. In Reference [24], the authors presented a novel algorithm that relied on learning automata to implement sleeping scheduling approaches, and it aimed to activate a minimum number of sensor nodes to achieve coverage of a certain area and guarantee connectivity between these nodes. Mukherjee et al. also focused on network coverage and connectivity issues, in Reference [25], they proposed a sleep-scheduling scheme that ensured a coverage degree requirement based on the dangerous levels of toxic gas leakage area, while maintaining global network connectivity with minimal awake nodes.

All these schemes can reduce the nodes’ energy consumption, but the working–sleeping cycle strategy for sensor nodes will cause opportunistic node connections, which can further result in the link instability. Therefore, modelling this kind of WSNs is a challenging task, and thus random graph theory was introduced in WSNs.

In Reference [26], the authors indicated that WSNs could be represented as a graph with a set of vertices consisting of the sensor nodes of the network and a set of edges consisting of the wireless links between the nodes, and they showed that it was appropriate to use a random geometric graph for modeling WSNs. The random geometric graph evolved from the random graph where edges only exist between adjacent nodes. In Reference [27], the authors further considered heterogeneous sensor nodes that may have different transmission ranges and designed a new routing metric, and then they proposed a novel random geometric graph model based on this metric for heterogeneous WSNs. In Reference [28], the authors focused on the weaknesses of modeling WSNs topology by the means of random graph theory, proposed a new weighted topology model for WSNs based on random geometric theory, in which the strength of the transmitted signal would decrease rapidly along the increasing of distance between different nodes was taken into consideration. In Reference [29], the authors proposed a model of WSNs on a two-dimensional plane using site percolation model, a kind of random graph in which edges are formed only between neighboring nodes, and based on which, they investigated WSNs connectivity and energy consumption at percolation threshold when a so-called phase transition phenomena happens. However, in some scenarios, it is not necessary to study the connectivity of the whole network. For example, in Reference [30], authors studied the problem of optimally determining source-destination connectivity of random networks, and by viewing the network as a random graph, they determined an optimal policy for establishing whether the designated source and destination were connected.

In this work, for any node who needs to send its source information that included in the probe message to the mobile sink, an energy efficient probe message forwarding mechanism is proposed. On receiving these probe messages and by analyzing the included source information, the mobile sink can determine the neighbor relationships of these nodes and calculate the link connectivity between any two adjacent nodes, and according to which, the mobile sink can construct an OCRG.

## 3. System Model

This section gives an analysis of the network model and the energy model used in this work.

### 3.1. Network Model

The network model used in this paper assumes that all sensor nodes are distributed in region Ω randomly and independently, and each node has a unique ID with radio range *r*. In addition, all nodes adopt asynchronous working–sleeping cycle strategy for sensing and communication, and the working time α and the sleeping time γ for each node can be anisochronous. Therefore, sensor nodes can communicate with each other only when they are in their working statuses. Moreover, this network supports multiple mobile sinks to collect data and each mobile sink has a unique Sink ID (SID) to distinguish from each other, however, we only use single mobile sink to describe the design principle in this work for simplicity. The mobile sink can move randomly and collect data actively anywhere in network at any time, it will always be in the working status for having sufficient energy and has two adjustable radio range *R_l_* and *R_s_*, of which *R_l_* is the long radio range that is fixed in each data collection period but adjustable in different periods used to broadcast the tag message to launch a data collection task, and *R_s_* is the short radio range that is always equal to *r* used to transfer data. The symbols and parameters used in network are summarized in Table 1.

**Figure 2:**
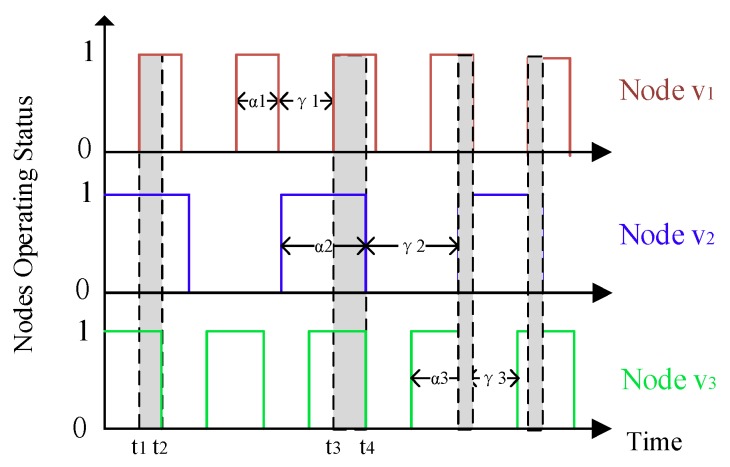
The asynchronous working–sleeping cycle strategy for sensor nodes: 0 for the sleeping status and 1 for the working status.

Figure 2 depicts the asynchronous working–sleeping cycle strategy for sensor nodes. Three given nodes $v_{1}$, $v_{2}$, $v_{3}$ are within the radio range of each other, but their operating statuses are asynchronous and their working time and sleeping time are anisochronous. Therefore, they can communicate with each other only when they are all in their working status, such as [t_1_,t_2_] or [t_3_,t_4_].

### 3.2. Energy Model

In this work, all sensor nodes adopt a simplified energy consumption model [31]. In the process of data collection, the energy consumption mainly consists of the received power and the transmission power. Here, let *ε_amp_* be the energy consumed in the transmission amplifier, *E_elec_* be the basic energy consumed during transmitting and receiving per bit respectively, and *d*_(*vi,vj*)_ be the distance between node *v_i_* and *v_j_*.

The received power by node *v_j_* that receives *B-*bit data is described as follows:

(1) $$E_{R}(v_{j})=E_{elec}B\;$$

The transmission power during node *v_i_* transmits *B-*bit data to node *v_j_* is described as follows:

(2) $$E_{T}(v_{i},v_{j})=(E_{elec}+\varepsilon_{amp}d_{\left(v_{i},v_{j}\right)}^{2})B\;$$

## 4. Data Collection Scheme Based on Opportunistic Node Connections

In the scheme, the mobile sink remains stationary and can determine the collection scope during the data collection. Once this data collection is completed, it can move in the network randomly and launch another data collection task anywhere in the network at any time. The proposed scheme can be presented in detail in the following four phases:

### 4.1. Initialization

To start a data collection period, the mobile sink can launch a data collection task anywhere in network at any time by broadcasting a tag message (including the duration for this data collection and the SID of the sink) by a long radio range *R_l_*, and then it should keep stationary until accomplish this data collection task. Therefore, all nodes under this radio range *R_l_* could receive this tag message.

On receiving a tag message, by obtaining the duration for this data collection included in this message, a node can calculate its whole working time (according to its own working–sleeping scheduling) and its status transition (from the working status to the sleeping status, or vice versa) frequency during this data collection period, which will be used to construct the OCRG in further.

For a node in current data collection scope, the method of Received Signal Strength Indication (RSSI) is used to estimate its distance to the mobile sink, the value of which is used to calculate its Expected Optimal Hops (EOH) (Definition 1) according to Lemma 1. The EOH of a node will be employed to select proper forwarders to minimize the overall energy consumption when forwarding the probe message to the mobile sink.

In order to facilitate the sleeping node to participate in data collection when it wakes up, the mobile sink can choose to re-broadcast the tag message randomly, and in this case, the nodes that have received the same tag message will not deal with it again.

**Definition** **1.** *Expected Optimal Hops (EOH). The Expected Optimal Hops denote the number of hops it takes to forward a probe message from any node to the mobile sink with the minimized energy consumption.*

It should be noted that EOH is different from the shortest path, according to the energy model, the energy consumption for a node to transfer data to the mobile sink is not linearly related to the length of the data transmission path.

**Lemma** **1.** *For any node v_i_ in a data collection scope, its EOH satisfies:*

(3) $${EOH}_{v_{i}}=\sqrt{\frac{\varepsilon_{amp}}{2E_{elec}}}d_{\left(v_{i},v_{\sin k}\right)}\;$$

*where d_(vi,vsink)_ is the distance between node v_i_ and the mobile sink.*

**Proof** **of** **Lemma** **1.** Assuming that there is *B*-bit data from node $v_{i}$ should be forwarded to the mobile sink *v*_sin*k*_ along the path (*v_i_*, *v*_sin*k*_, *k*), where *k* is the hops on the path and satisfies 1 ≤ *k* ≤ N − 1. To unify the path representation, *v*_sin*k*_ is represented by *v_i+k_* in the next proof process, from which it means that it needs *k* hops to forward a probe message from *v_i_* to *v*_sin*k*_. Then, the energy consumption function with respect to *k* for this forwarding can be calculated as follows:

(4) $$\begin{array}{cc} C_{v_{i}}(k) & =\sum_{j=0}^{k-1}\left(E_{T}+E_{R}\right)\\ & =\sum_{j=0}^{k-1}\left\{\left[E_{elec}+\varepsilon_{amp}d_{\left(v_{i+j},v_{i+(j+1)}\right)}^{2}\right]B+E_{elec}B\right\}\\ & =(2k)E_{elec}B+\varepsilon_{amp}\left[\sum_{j=0}^{k-1}d_{\left(v_{i+j},v_{i+(j+1)}\right)}^{2}\right]B \end{array}$$

To get the minimum value of *C_vi_*(*k*), we can use the average value inequality to derive inequality of *C_vi_*(*k*) which can be described as follows:

(5) $$C_{v_{i}}(k)\geq (2k)E_{elec}B+\frac{\varepsilon_{amp}{\left[\sum_{j=0}^{k-1}d_{\left(v_{i+j},v_{i+(j+1)}\right)}\right]}^{2}B}{k}\;$$

Since the sum of the distances for any two adjacent nodes on the path (*v_i_*, *v_i_*_+*k*_, *k*) satisfies the following inequality:

(6) $$\sum_{j=0}^{k-1}d_{\left(v_{i+j},v_{i+(j+1)}\right)}\geq d_{\left(v_{i},v_{i+k}\right)}\;$$

Therefore, the minimum energy consumption function $C_{v_{i}}^{\min}\left(k\right)$ with respect to *k* satisfies:

(7) $$C_{v_{i}}^{\min}(k)=(2k)E_{elec}B+\frac{\varepsilon_{amp}d_{\left(v_{i},v_{i+k}\right)}^{2}B}{k}\;$$

To get the value of *k* that can minimize $C_{v_{i}}^{\min}\left(k\right)$, we can take the first derivative of $C_{v_{i}}^{\min}\left(k\right)$ with respect to *k* as follows:

(8) $$\frac{\partial C_{v_{i}}^{\min}}{\partial k}=2E_{elec}B-\frac{\varepsilon_{amp}d_{\left(v_{i},v_{i+k}\right)}^{2}B}{k^{2}}\;$$

Let ∂Cvimin∂k equal to 0, we can get a value of *k*. By using *k*_expected_ to denote this value, we have:

(9) $$k_{\mathrm{expected}}=\sqrt{\frac{\varepsilon_{amp}}{2E_{elec}}}d_{\left(v_{i},v_{i+k}\right)}\;$$

Next, taking the second derivative of $C_{v_{i}}^{\min}\left(k\right)$ with respect to *k* and comparing the value of it at *k = k*_expected_ with 0 as follows:

(10) $$\frac{\partial^{2}C_{v_{i}}^{\min}}{\partial k^{2}}{|}_{{k=k}_{\exp exted}}=\frac{\varepsilon_{amp}d_{\left(v_{i},v_{i+k}\right)}^{2}B}{k^{3}}{|}_{{k=k}_{\exp exted}}>0\;$$

Therefore, the $C_{v_{i}}^{\min}\left(k\right)$ has a minimum value at *k* = $k_{\mathrm{expected}}$, and since *v_i+k_* represents *v*_sin*k*_, so we here define the EOH for node *v_i_* is:

(11) $${EOH}_{v_{i}}=\sqrt{\frac{\varepsilon_{amp}}{2E_{elec}}}d_{{(v}_{i},v_{\sin k})}\;$$

The proof is completed. □

### 4.2. Energy Efficient Probe Message Forwarding Mechanism

For each node in the data collection scope, it needs to send its source information (including its whole working time, its status transition frequency and all its neighbors’ IDs) included in the probe messages to the mobile sink. The format of the probe message is given in Table 2.

The “Message Source” field is used to indicate the ID of the node sending this probe message. The “Source Information” field is used to store the whole working time, the status transition frequency, and the neighbors’ IDs of the message source. The “Sink ID” field indicates the SID of the mobile sink launching this data collection task, and which is also the forwarded destination for the probe messages. The “Forwarders ID” field is used to store the IDs of nodes that have forwarded this probe message in sequence. In the process of forwarding a probe message, each time the message is relayed by a node (forwarder), it should add its ID in the “Forwarders ID” field in the probe message. The “EOH” field is used to store the value of EOH for the message source.

Since a probe message can be generated from any node that received a tag message and the opportunistic node connections in the network decrease the efficiency of probe message forwarding, it is necessary to design an energy efficient probe message forwarding mechanism to minimize the overall energy consumption when forwarding them to the mobile sink.

In the mechanism, only the nodes that have received this tag message can participate in the probe message forwarding. When an intermediate node *v_m_* receives a probe message, it will check the IDs stored in “Forwarders ID” field in this probe message to learn whether it has forwarded it or not.

**Figure 3:**
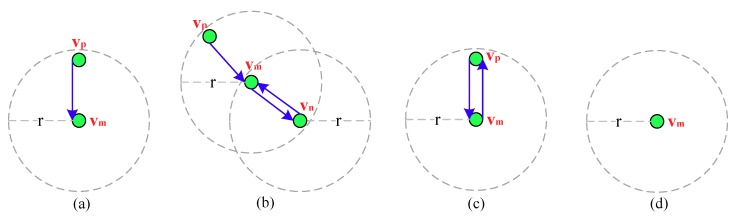
The illustration of four cases when *v_m_* receives the probe message, (**a**) *v_m_* receives the probe message for the first time; (**b**) *v_m_* has forwarded this message; (**c**) only the neighbor *v_p_* of *v_m_* is in working status; (**d**) *v_m_* has no neighbor.

Here if *v_m_* has not forwarded this message, as shown in Figure 3a, it first updates the “Forwarders ID” field by adding its ID in it and calculates the Number of Forwarders (NF) based on it, by obtaining the value of EOH in this message it then can further calculate (EOH–NF), to find a neighbor whose EOH is closest to it as an optimal forwarder. However, if *v_m_* has forwarded this message, as shown in Figure 3b, for sake of exposition, suppose that the IDs in the “Forwarders ID” field are denoted as {$v_{s}$,…, $v_{p}$, $v_{m}$, $v_{n}$}, it first explores the neighbors that have not forwarded this probe message by checking the IDs in this field, and then updates this field by deleting the IDs of *v_m_*, *v_n_* from it and adds its ID in it. In the next, by calculating NF based on this field and obtaining the value of EOH in this message, *v_m_* can further calculate (EOH–NF) to find a neighbor whose EOH is closest to it from the explored neighbors as an optimal forwarder.

Considering the asynchronous working–sleeping cycle strategy for nodes, when *v_m_* receives this probe message from *v_p_*, if only the neighbor *v_p_* is in working status, as shown in Figure 3c, it forwards the message to *v_p_*. However, if *v_p_* is also in sleeping status, as shown in Figure 3d, *v_m_* stops forwarding it. Algorithm 1 gives the detailed energy efficient probe message forwarding mechanism.

**Algorithm 1.** Energy efficient probe message forwarding mechanism
**1.** When an intermediate node *v_m_* receives a probe message:**2.** ***if*** (*v_m_* has not forwarded the probe message)**3.** Add its ID in the “Forwarders ID” field included in the probe message;**4.** Calculate NF based on the “Forwarders ID” field;**5.** Calculate (EOH–NF);  // *the EOH is obtained from the “EOH” field***6.** ***if*** (*v_m_* has neighbors)  //*v_p_ is not included***7.** Select the neighbor whose EOH is closest to (EOH–NF) as an optimal forwarder;**8.** Forward the probe message to it;**9.** 
***end if***
**10.** 
***else***
**11.** //*suppose that v_m_ has forwarded the probe message to v_n_, but then receives this message again from it***12.** Explore the neighbors that have not forwarded this probe message;**13.** Delete the IDs of *v_m_*, *v_n_* from the “Forwarders ID” field;**14.** Node *v_m_* adds its ID in the “Forwarders ID” field;**15.** Calculate NF based on the “Forwarders ID” field;**16.** Calculate (EOH–NF);**17.** ***if*** (the explored neighbors exist)**18.** Select the neighbor whose EOH is closest to (EOH–NF) as an optimal forwarder;**19.** Forward the probe message to it;**20.** 
***end if***
**21.** 
***end if***
**22.** ***if*** (only the neighbor *v_p_* of *v_m_* is in working status)**23.** Forward the probe message to *v_p_*;**24.** ***else***  //*v_m_ is in sleeping status***25.** Stop forwarding this probe message;**26.** 
***end if***



### 4.3. Opportunistic Connection Random Graph Construction

After receiving these probe messages, the mobile sink constructs an OCRG (Definition 2) by analyzing the included source information to reflect the opportunistic node connections in this data collection scope.

**Definition** **2.** *Opportunistic Connection Random Graph (OCRG). The OCRG is constructed based on the intermittent communication caused by asynchronous working–sleeping cycle strategy of nodes, which can be analyzed from all the source information, to reflect the opportunistic node connections in the data collection scope, denoted as G(V, E, P_e_).*

In an OCRG, V denotes a set of all message sources, E denotes a set of all opportunistic connections between any two adjacent nodes in set V, and *P_e_* denotes the link connectivity between any two adjacent nodes in set V.

**Figure 4:**
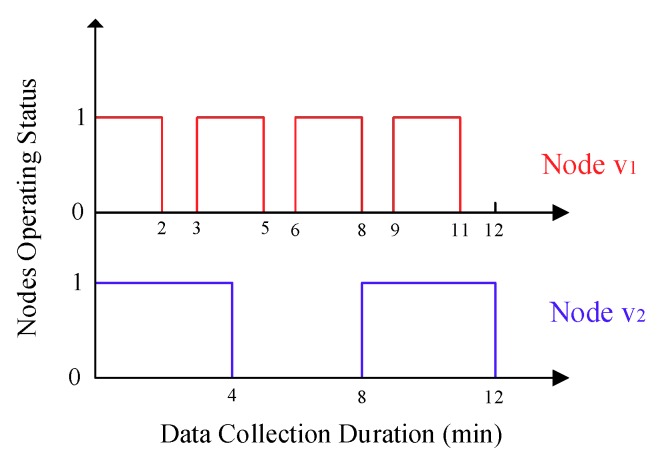
Comparison of the asynchronous working–sleeping cycle strategy for different nodes, where 0 represents the sleeping status and 1 represents the working status.

For sake of exposition, the link connectivity *P_e_* between any two adjacent nodes *v_i_* and *v_j_* is denoted as $P_{v_{i}v_{j}}$. There are two factors affect the value of $P_{v_{i}v_{j}}$, of which the first one is the whole working time of nodes *v_i_* and *v_j_*, the longer the nodes’ whole working time, the more likely they can communicate with each other. In addition, the second one is the status transition frequencies of nodes *v_i_* and *v_j_*, which can be explained by that, under the same duration for a data collection, sensor nodes with different status transition frequencies may produce the same whole working time, but nodes with higher status transition frequencies contribute more to improve the link connectivity. This phenomenon can be illustrated in Figure 4, in which the duration for a data collection for the mobile sink is 12 min, and the working–sleeping cycle for node $v_{1}$ and $v_{2}$ is 3 min and 8 min, respectively, so both of them work 8 min during this process, but the status transition frequency for node $v_{1}$ is about 2.3 times that of node $v_{2}$.

Based on the analysis above, the link connectivity ($P_{v_{i}v_{j}}$) between any two adjacent nodes *v_i_* and *v_j_* can be calculated as follows:

(12) $$P_{v_{i}v_{j}}={f_{i}p}_{v_{i}}\times f_{j}p_{v_{j}}=\left(\frac{f_{v_{i}}}{f_{\max}}\times \frac{T_{v_{i}}}{T_{p}}\right)\left(\frac{f_{v_{j}}}{f_{\max}}\times \frac{T_{v_{j}}}{T_{p}}\right)\;$$

where $T_{v_{i}}$ and $T_{v_{j}}$ is the whole working time of node *v_i_* and *v_j_* in a data collection period $T_{p}$, respectively, while $f_{v_{i}}$ and $f_{v_{j}}$ is the status transition frequencies of node *v_i_* and *v_j_* in this period, respectively. Here $f_{\max}$ is the max value of the status transition frequency obtained from all the probe messages, and it is used to achieve normalization of the node’s status transition frequency.

Noted that the mobile sink is always in working status, any of its adjacent node (under its short radio range $R_{s}$) that in working status can communicate with it at any time. Hence, for the mobile sink and any of its adjacent node *v_i_*, their link connectivity ($P_{v_{i}v_{\sin k}}$) can be affected by the whole working time and the status transition frequency of node *v_i_*, and accordingly, $P_{v_{i}v_{\sin k}}$ can be calculated as follows:

(13) $$P_{v_{i}v_{\sin k}}={f_{i}p}_{v_{i}}\times P_{v_{\sin k}}=\frac{f_{v_{i}}}{f_{\max}}\times \frac{T_{v_{i}}}{T_{p}}\;$$

By combing Equations (12) and (13), *P_e_* can be described as follows:

(14) $$P_{e}=\{\begin{array}{l} \frac{f_{v_{i}}}{f_{\max}}\times \frac{T_{v_{i}}}{T_{p}},\begin{array}{c} P_{v_{i}v_{\sin k}} \end{array}\\ \left(\frac{f_{v_{i}}}{f_{\max}}\times \frac{T_{v_{i}}}{T_{p}}\right)\left(\frac{f_{v_{j}}}{f_{\max}}\times \frac{T_{v_{j}}}{T_{p}}\right),\begin{array}{l} P_{v_{i}v_{j}} \end{array} \end{array}\;$$

**Figure 5:**
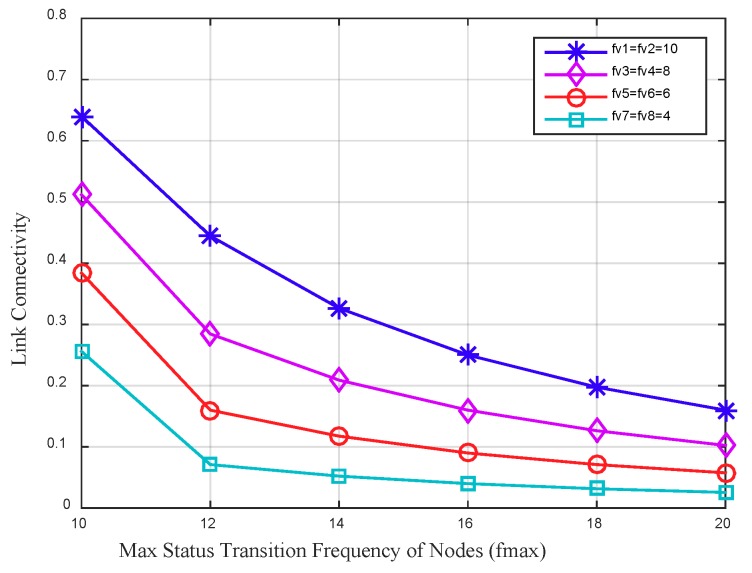
Link connectivity between four node pairs ($v_{1}$ is adjacent to $v_{2}$, $v_{3}$ is adjacent to $v_{4}$, $v_{5}$ is adjacent to $v_{6}$, $v_{7}$ is adjacent to $v_{8}$) varies by the max status transition frequency of nodes, in which the whole working time of nodes are set to 80 percent of $T_{p}$.

Figure 5 depicts the link connectivity between four node pairs varies by the max status transition frequency of nodes. The whole working time of nodes in this data collection period is set to a fixed value to explore the impact of different status transition frequency of nodes on the link connectivity. In the figure it can be seen that, with a fixed whole working time of the node, the link connectivity between nodes decreases as the max status transition frequency of nodes increases. For two adjacent nodes with higher status transition frequency, the link connectivity between them is higher. The main reason is that the higher status transition frequency of a node increases the communication opportunities with its neighbors, and further improves the link connectivity between them.

Based on the analysis above, the mobile sink can first obtain the message source set V by gathering their IDs stored in the “Message Source” field included in the probe messages, and then obtain the edge set E by analyzing the neighbors’ IDs of each message source that stored in the “Source Information” field, by gathering all message sources’ whole working time and status transition frequencies stored in the “Source Information” field it can calculate the link connectivity between any two adjacent nodes in set V according to Equation (14), thus constructing an OCRG.

By constructing OCRG, the mobile sink models the opportunistic node connections in the data collection scope, based on which, it could calculate the optimal data forwarding path in advance for each node to send their sensing data to improve the reliability of data transmission. Furthermore, the source node could obtain its data forwarding path in advance therefore reduce the delay in each hop, and due to the intermittent communication mode of the nodes, it could also improve the success rate of the data transmission.

**Figure 6:**
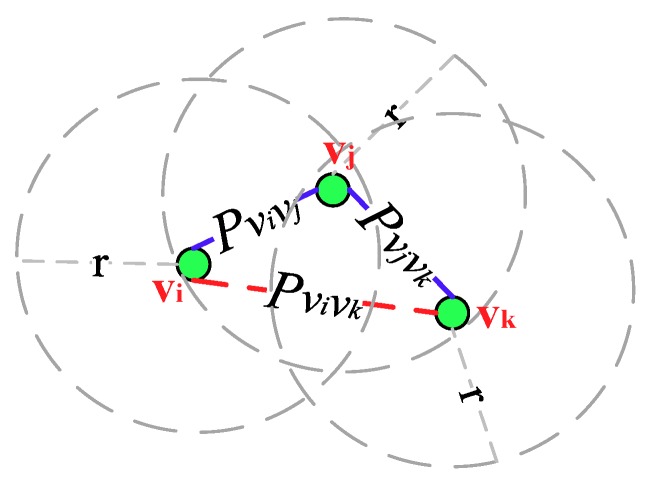
An illustration of an Opportunistic Connection Random Graph (OCRG) with three nodes.

Figure 6 depicts an OCRG with three nodes *v_i_*, *v_j_* and *v_k_* that adopt the asynchronous working–sleeping cycle strategy to save energy. Nodes *v_i_* and *v_j_* are adjacent neighbors while *v_j_* and *v_k_* are adjacent neighbors, and the link connectivity between them are $P_{v_{i}v_{j}}$ and $P_{v_{j}v_{k}}$, respectively, hence there exists an opportunistic connection. Since node *v_i_* is not adjacent to *v_j_*, their link connectivity P_*v_i_v_k_*_ is 0.

### 4.4. The Optimal Path Calculation Based on the Opportunistic Connection Random Graph

Once the OCRG is constructed, it needs to find the optimal path from any node to the mobile sink to realize the reliable sensing data forwarding. In this work, a spanning tree algorithm (Algorithm 2) is designed to calculate the optimal path with the max value of Path Connectivity (PC) (Definition 3) from the mobile sink to each node based on this OCRG. By employing this algorithm, the mobile sink can produce a spanning tree, based on which, each node can get its optimal path to the sink with the corresponding PC of it. Therefore, the mobile sink will broadcast this spanning tree to nodes under the long radio range $R_{l}$.

**Definition** **3.** *Path Connectivity (PC). In the OCRG, the PC of a path is related to the link connectivity between any two adjacent nodes on this path, which is used to reflect the reliability of this path when delivering data. As for a path ($v_{i}$, $v_{i+k}$, k), where node $v_{i}$ is the source node, node $v_{i+k}$ represents the node k hops away from $v_{i}$ on this path, k is the hops on the path and satisfies 1 ≤ k ≤ N − 1, the PC of this path is defined as:*

(15) $${PC}_{path(v_{i},v_{i+k},k)}=\prod_{j=0}^{k-1}P_{v_{i+j}v_{i+(j+1)}}\;$$

During constructing this spanning tree, all nodes in the OCRG are subdivided into two sets: (i) set K: including the nodes whose optimal paths from the mobile sink to themselves are known, and (ii) set U: including the remaining nodes. In the beginning, set K includes only the mobile sink while set U includes the remaining nodes in the OCRG, and a node in set U will be transferred to set K if the mobile sink finds the optimal path to it.

The algorithm includes two phases, of which the first phase is initialization. In this phase, the mobile sink calculates the PC of the paths from itself to all nodes in set U as follows:

(16) $${PC}_{path(v_{\sin k},v_{i},*)}=\left\{ \begin{array}{ll} P_{v_{\sin k}v_{i}} & ,v_{\sin k}\;is\;directly\;connected\;to\;v_{i}\\ 0 & ,otherwise \end{array}\right.$$

And the nodes on each path can be denoted as follows:

(17) $$PATH(v_{\sin k},v_{i},*)=\left\{ \begin{array}{ll} \left\{v_{\sin k},v_{i}\right\} & ,v_{\sin k}\;is\;directly\;connected\;to\;v_{i}\\ \emptyset & ,otherwise \end{array}\right.$$

In the second phase, the mobile sink selects a node $v_{i}$ with a max value of PC from set U and transfers it to set K, and then updates the PC of the path from $v_{\sin k}$ to each node $v_{j}$ in set U but directly connected to $v_{i}$ as follows:

(18) $${PC}_{path(v_{\sin k},v_{j},*)}\leftarrow \max\left\{{PC}_{path(v_{\sin k},v_{j},*)},{PC}_{path(v_{\sin k},v_{i},*)}\times P_{v_{i}v_{j}}\right\}\;$$

For each path, once the PC of it is updated, the nodes on it should be updated as follows:

(19) $$PATH(v_{\sin k},v_{j},*)\leftarrow PATH(v_{\sin k},v_{i},*)+v_{j}\;$$

Repeating the work in second phase until set U is empty, a spanning tree with the mobile sink as the root node can be generated.

The algorithm improves the efficiency of finding the optimal path. If the mobile sink calculates and selects the optimal path of each node directly, for each node, it needs to explore all the possible paths between them to determine which one has the maximum PC, which is inefficient and brings high delay. Furthermore, when the network density increases or the data collection scope expands, the number of the paths from a node to the mobile sink increases greatly, which could further decrease its efficiency.

**Algorithm 2.** The spanning tree algorithm
**1.** **Input:** an OCRG;**2.** **Output:** a spanning tree with the mobile sink as the root node;**3.** Add $v_{\sin k}$ into set K;**4.** Add the remaining nodes in the OCRG into set U;**5.** ***for*** each node $v_{i}$ in set U ***do*** //  *initialization***6.** ***if*** ($v_{i}$ is directly connected to $v_{\sin k}$)**7.** ${PC}_{path(v_{\sin k},v_{i},*)}$ = $P_{v_{\sin k}v_{i}}$;   //${PC}_{path(v_{\sin k},v_{i},*)}$ *is the PC of the path from* $v_{\sin k}$ *to* $v_{i}$**8.** $PATH(v_{\sin k},v_{i},*)$ = {$v_{\sin k}$,$v_{i}$}; //$PATH(v_{\sin k},v_{i},*)$ *is used to store the nodes on the path from* $v_{\sin k}$ *to* $v_{i}$**9.** 
***else***
**10.** ${PC}_{path(v_{\sin k},v_{i},*)}$ = 0;**11.** $PATH(v_{\sin k},v_{i},*)$ = ∅;**12.** 
***end if***
**13.** 
***end for***
**14.** ${PC}_{path(v_{\sin k},v_{\sin k},*)}$ = 1;    // *set the PC of the path from* $v_{\sin k}$ *to* $v_{\sin k}$ is 1**15.** ***while*** (set U is not empty)**16.** Select the node $v_{i}$ with max ${PC}_{path(v_{\sin k},v_{i},*)}$ from set U;**17.** Transfer $v_{i}$ from set U to set K;**18.** ***for*** each neighbor $v_{j}$ of $v_{i}$ ***do***    //$v_{j}$ *is still in set U***19.** temp ←${PC}_{path(v_{\sin k},v_{i},*)}$×$P_{v_{i}v_{j}}$;**20.** ***if*** (temp > ${PC}_{path(v_{\sin k},v_{j},*)}$)**21.** ${PC}_{path(v_{\sin k},v_{j},*)}$← temp;  // *update the PC of the path from* $v_{\sin k}$ *to* $v_{j}$**22.** $PATH(v_{\sin k},v_{j},*)$←$PATH(v_{\sin k},v_{i},*)$ + $v_{j}$; // *update the nodes on the path from* $v_{\sin k}$ *to* $v_{j}$**23.** 
***end if***
**24.** 
***end for***
**25.** 
***end while***



**Figure 7:**
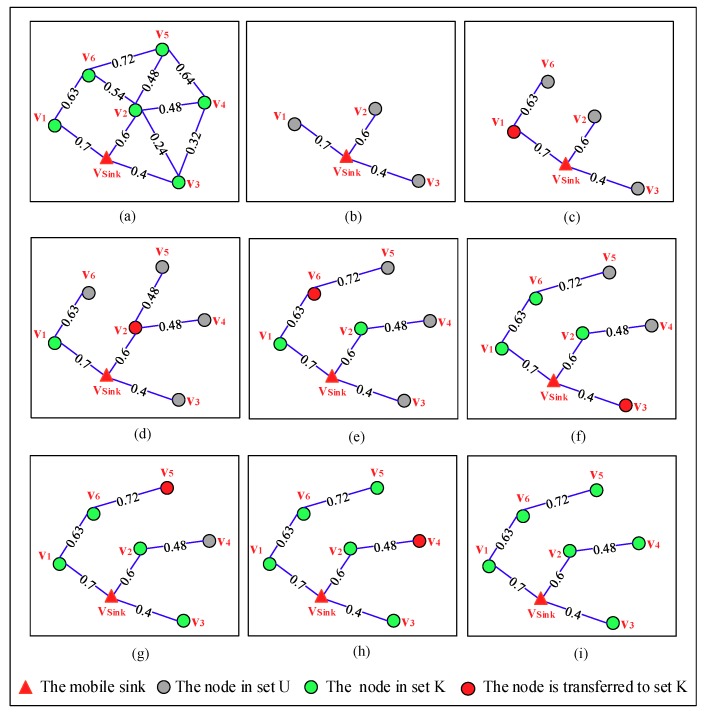
The process of constructing the spanning tree step by step, in which a node with max value of PC in set U will be transferred to set K, (**a**) an OCRG with seven nodes; (**b**) initialization; (**c**) step 1: transferring $v_{1}$; (**d**) step 2: transferring $v_{2}$; (**e**) step 3: transferring $v_{6}$; (**f**) step 4: transferring $v_{3}$; (**g**) step 5: transferring $v_{5}$; (**h**) step 6: transferring $v_{4}$; (**i**) a spanning tree with $v_{\sin k}$ as the root node.

Figure 7 depicts the detailed process of constructing the spanning tree step by step in an OCRG with seven nodes. From Figure 7a we can see, sets K and U can be denoted as {$v_{\sin k}$} and {$v_{1}$,$v_{2}$,$v_{3}$,$v_{4}$,$v_{5}$,$v_{6}$}, respectively. In the first phase of the algorithm,$v_{\sin k}$ calculates the PC of the paths from itself to all nodes in set U and finds the nodes on each path, the results of which are shown in Table 3 and Figure 7b, respectively. The PC[$v_{i}$] (*i* = 1, 2,…, 6) used in this table indicates the PC of the path from the mobile sink to node $v_{i}$, and the value with bold format is the max value of PC in set U. The second phase of this algorithm includes 6 steps, which can be seen from Figure 7c–h. In step 1, node $v_{1}$ is transferred to set K due to it has the max value of PC among that of all nodes in set U, which can be seen in the table. By checking the OCRG, $v_{\sin k}$ learns that only $v_{6}$ in set U is directly connected to $v_{1}$, so it updates the PC of the path from itself to $v_{6}$ and the nodes on this path. In the next 5 steps, by repeating the work in step 1, a spanning tree is generated which can be seen in Figure 7i.

When the spanning tree is generated, the mobile sink should broadcast it to the nodes under its long radio range $R_{l}$ so that they can forward the sensing data to it according to this spanning tree. On receiving the spanning tree, here it is noticing that, only a node that is on it could obtain the optimal path from itself to the mobile sink, and store the path and the PC of the path locally. However, if a node does not find itself on the spanning tree, it will re-send its probe message to the mobile sink according to the energy efficient probe message forwarding mechanism. Moreover, recalling that the mobile sink can choose to re-broadcast the tag message randomly during the current data collection period, the nodes that have not received this tag message will also send their probe messages to the mobile sink.

On receiving these probe messages, the mobile sink could re-construct an OCRG, then re-calculate the optimal path from itself to each node in this OCRG and update the spanning tree accordingly.

In fact, for a node, its optimal path on the spanning tree is the most reliable path for it to forward its sensing data to the mobile sink. However, due to using the asynchronous working–sleeping cycle strategy for nodes, so during data transmission process along this path, any forwarder on the path may be in sleeping status which could lead to the failed link connection, it further causes the failure of data transmission. To solve this problem, a routing protocol that adapts to different nodes operating statuses is proposed to improve the reliability of data transmission and Algorithm 3 gives the detailed routing protocol. In this routing protocol, assuming that the sensing data from node $v_{s}$ is being forwarded along its optimal path on the spanning tree to the mobile sink. When an intermediate node $v_{m}$ receives the data, it should forward it to its father node $v_{n}$ only if $v_{n}$ is in working status, otherwise, $v_{m}$ should select other forwarder from its neighbors to continue this data forwarding. This kind of forwarder selection method is described as follows.

For each neighbor $v_{i}$ of $v_{m}$ that is on the spanning tree, $v_{m}$ calculates the link connectivity $P_{v_{m}v_{i}}$ between them according to Equation (14), by obtaining the value of PC (${PC}_{path(v_{i},v_{\sin k},*)}$) stored in $v_{i}$ locally it then can calculate ($P_{v_{m}v_{i}}$×${PC}_{path(v_{i},v_{\sin k},*)}$) to get the PC of the path $v_{m}$-$v_{i}$*-*$v_{\sin k}$, accordingly, $v_{m}$ selects the neighbor on path $v_{m}$-$v_{i}$*-*$v_{\sin k}$ with a max value of PC as the forwarder.

**Algorithm 3.** The routing protocol
**1.** Assuming that the sensing data from node $v_{s}$ is forwarded along its optimal path on the spanning tree to the mobile sink;**2.** When an intermediate node $v_{m}$ receives the sensing data:**3.** ***if*** (its father node $v_{n}$ is in working status)**4.** Forward the sensing data to $v_{n}$;**5.** $v_{n}$ employs this routing protocol to forward the data;**6.** ***else***      //$v_{n}$*is in sleeping status***7.** ***if*** ($v_{m}$ has neighbors on the spanning tree)**8.** ***for*** each neighbor $v_{i}$ ***do*****9.** Calculate $P_{v_{m}v_{i}}$ according to Equation (14);**10.** Calculate the PC of the path $v_{m}$-$v_{i}$-$v_{\sin k}$;**11.** 
***end for***
**12.** Select the neighbor that on a path with a max value of PC as the forwarder;**13.** Forward the sensing data to this forwarder;**14.** The forwarder employs this routing protocol to forward the data;**15.** 
***else***
**16.** Stop forwarding this data;**17.** 
***end if***
**18.** 
***end if***



**Figure 8:**
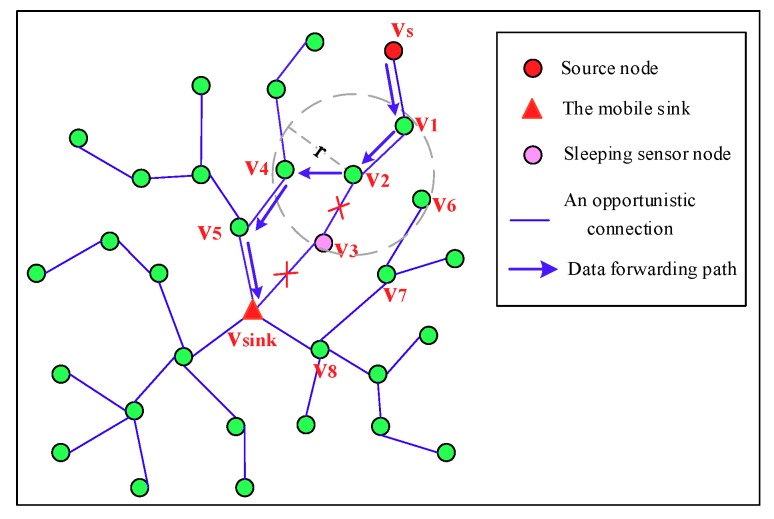
The process of a source node $v_{s}$ forwards its sensing data to the mobile sink along the optimal path on the spanning tree.

Figure 8 depicts the detailed process of a source node $v_{s}$ forwards its sensing data to $v_{\sin k}$. Node $v_{s}$ forwards the data to its father node $v_{1}$, and then $v_{1}$ forwards it to its father node $v_{2}$. Since the father node $v_{3}$ of $v_{2}$ is in sleeping status, $v_{2}$ needs to find other forwarder. For neighbors $v_{4}$ and $v_{6}$ of $v_{2}$ that are on the spanning tree, $v_{2}$ calculates link connectivity $P_{v_{2}v_{4}}$ and $P_{v_{2}v_{6}}$, respectively, by obtaining the value of PC stored in $v_{4}$, $v_{6}$ locally it then can calculate ($P_{v_{2}v_{4}}$×${PC}_{path(v_{4},v_{\sin k},*)}$) and ($P_{v_{2}v_{6}}$×${PC}_{path(v_{4},v_{\sin k},*)}$), respectively, to get the PC of the path $v_{2}$-$v_{4}$-$v_{5}$*-*$v_{\sin k}$ and path $v_{2}$-$v_{6}$-$v_{7}$-$v_{8}$*-*$v_{\sin k}$. Since the PC of the path $v_{2}$-$v_{4}$-$v_{5}$*-*$v_{\sin k}$ is greater than that of the path $v_{2}$-$v_{6}$-$v_{7}$-$v_{8}$*-*$v_{\sin k}$, node $v_{4}$ is selected as the forwarder, then the sensing data is forwarded to $v_{\sin k}$ along the path $v_{4}$-$v_{5}$*-*$v_{\sin k}$.

## 5. Performance Evaluation

### 5.1. Simulation Environment

Our simulations are performed under Matlab R2014b simulator [32] and the parameters used in this simulation are listed in Table 4.

In this simulation, sensor nodes are deployed in a 400 × 400 m^2^ region randomly and independently. The random mobility mode has two stages: mobile and stationary, and the mobile sink can independently determine the direction and speed when moving, and it can decide the dwell time when keeping stationary. The duration for a data collection period varies and the mobile sink can change it in different collection tasks, but in our experiments, we fix this value to compare the impact of the other parameters on the performance of the algorithm. For classical data transmission schemes, in ExOR [33], by taking advantages of the broadcast nature of the wireless medium and allowing multiple neighbors that overhear the transmission to participate in forwarding data, it can improve the reliability of data forwarding. In Probabilistic and Opportunistic Flooding Algorithm (POFA) [34], by adopting the controlled transmissions, it can achieve a target reliability on each hop of data forwarding. In Reference [35], a data transmission schemes named Reliable Proliferation Routing with low Duty Cycle (RPRDC) is proposed, by integrates three core concepts namely reliable path finder, a randomized dispersity, and forwarding, it improves the success rate of packet forwarding. In addition, both of them consider unreliable wireless links when making the routing decision. In this work, the performance of the proposed scheme is compared with that of ExOR, POFA and RPRDC in terms of the determined performance metrics, (i) Packet Delivery Ratio (PDR), (ii) Energy Consumption (EC), and (iii) Network Lifetime (NL).

### 5.2. Evaluation Results

#### 5.2.1. Packet Delivery Ratio

**Figure 9:**
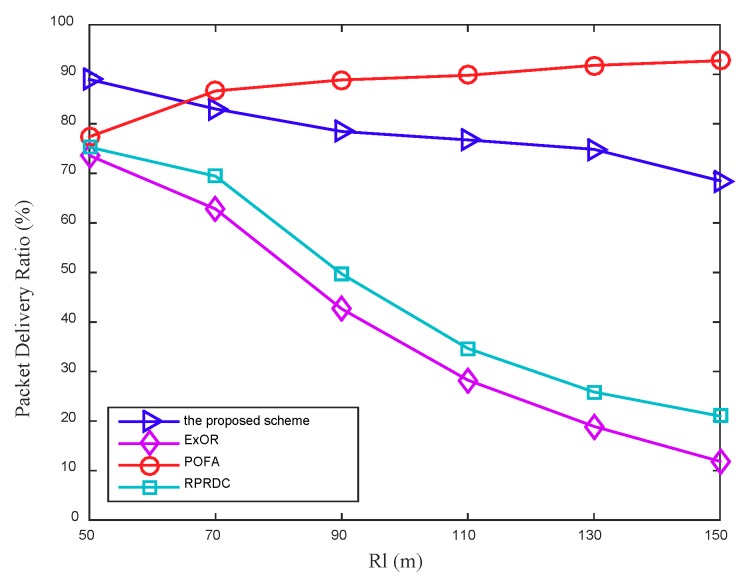
The Packet Delivery Ratio (PDR) varies with the long radio range $R_{l}$ of the mobile sink, in addition, the number of nodes (N) is 1100.

In this work, the PDR is defined as the ratio of packets received by the mobile sink to packets sent by the sensor nodes, and three scenarios are designed to conduct simulations to evaluate the PDR based on the long radio range $R_{l}$ of the mobile sink and the network density. In these experiments, we consider two cases that a node will drop the data packet, first, a node cannot find a forwarder due to the asynchronous working–sleeping cycle strategy for sensor nodes. Second, when data packets are forwarded between two adjacent nodes, a node enters sleeping status and causes transmission failure. As shown in Figure 9, the PDR in POFA increases as the long radio range $R_{l}$ of the mobile sink increases while it decreases in the proposed scheme, ExOR and RPRDC. The reason is that when $R_{l}$ increases, more nodes under the data collection scope will join the data collection process, and due to the network density keeps unchanged, the number of hops these newly joined nodes takes to forward their sensing data to the mobile sink increases correspondingly, which decreases the success rate of delivering the sensing data in the proposed scheme, ExOR and RPRDC. However, in POFA, the sensing data of a node will be forwarded along multiple paths until one of them connected to the mobile sink, as the number of nodes in this scope increase, the number of the possible paths increases, which improves the success rate of data delivering.

From the figure it can be seen that, the proposed scheme performs better PDR (almost one time and 72%) than that in ExOR and RPRDC as $R_{l}$ expands, the reason is that, on one hand, a node in the proposed scheme always forwards its sensing data along its optimal path that keeps the maximum PC. On the other hand, the routing protocol in the scheme can adjust the optimal path when it is disconnected due to node sleeping, which can further improve its PDR.

**Figure 10:**
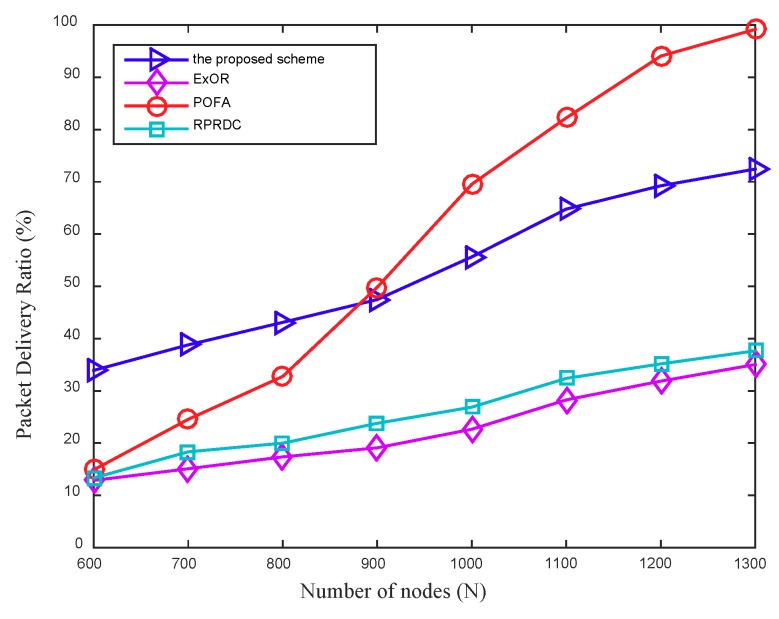
The PDR varies with the network density, in addition, $R_{l}$ = 110 m.

As shown in Figure 10, the PDR increases as the network density increases, the proposed scheme achieves almost 130% and 102% improvement in PDR over ExOR and RPRDC, respectively. Since the number of working nodes in network increases with the increased network density, it can improve the network connectivity clearly and therefore increase the overall PDR.

The proposed scheme outperforms POFA when the number of nodes in network is less than 900, but as the network density increases continuously, POFA performs better. This can be explained by that in POFA, a node needs to select multiple forwarders to achieve its target reliability, and the improvement of network connectivity increases the possibility of achieving this target reliability rapidly, which further improves its PDR. The main reason that the proposed scheme is always performs better than ExOR and RPRDC can be explained by the different routing strategies they employed. In RPRDC, the routing is divided into random dispersion and reliability path exploration, of which the packet reception rate, residual energy, and link quality of the node are considered, but it lacks the global information between the node and the sink. Furthermore, ExOR adopts the metric ETX associated with the link connectivity in network, to reflect the expected transmission times required to forward a data from a node to the mobile sink, and it selects a node with a minimum value of ETX as the forwarder during the data forwarding. However, in the proposed scheme, the metric PC reflects the connectivity of a path directly and the routing protocol can guarantee the data always be forwarded along a path with a max value of PC, so it achieves a higher success rate of data delivering than that in ExOR and RPRDC.

In the next scenario, we conduct a simulation to evaluate the performance of these four algorithms in PDR with a large enough data collection scope, in which the mobile sink keeps stationary in the center of network and the long radio range $R_{l}$ of the mobile sink is set to 283 m so that it can cover the whole network.

**Figure 11:**
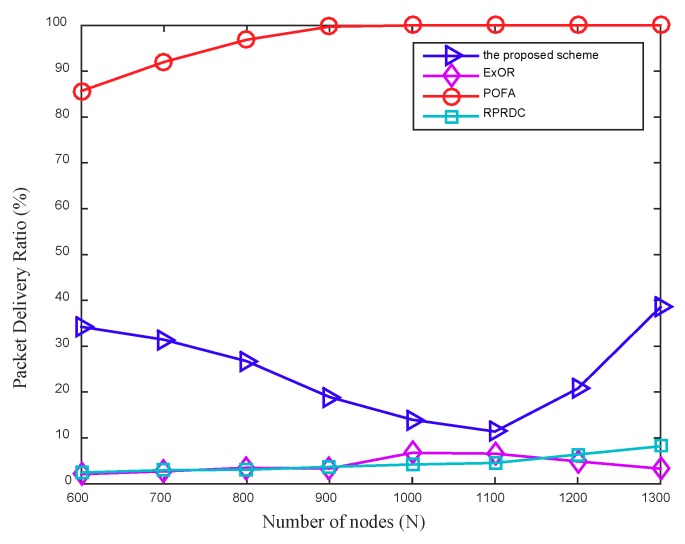
The PDR varies with the network density, in addition, $R_{l}$ = 283 m.

Figure 11 shows that as the network density increases, the PDR in the proposed scheme first decreases and then increases. The main reason is that increasing the number of nodes not only improves the network connectivity, but also increases the number of packets sent by source nodes, when the number of nodes in network increases from 600 to 1100, the increased packets sent by the source nodes decreases its PDR, however, as the increased number of nodes is greater than 1100, the improved network connectivity increases its PDR.

The PDR in POFA is always higher than that in the proposed scheme, ExOR and RPRDC, which can be explained by that, in POFA, this large data collection scope increases the number of possible paths for a source node to send its sensing data to the mobile sink, which improves the success rate of data delivering greatly. However, in the proposed scheme, ExOR and RPRDC, this large scope increases the number of hops the nodes that near the edge of the data collection scope takes to forward their sensing data, which decreases the success rate of data delivering greatly. In addition, the proposed scheme achieves a better performance in PDR than that in ExOR and RPRDC. Since the node adopts the intermittent communication mode, the delay of data delivering in each hop will affect the success rate of this forwarding, especially when the node is far away from the mobile sink. In ExOR, a node needs to run a protocol to explore the neighbors that received the data and from which to select forwarders, therefore it delays the data forwarding and then decreases the success rate of data delivering. During the reliable path exploration phase of RPRDC, a node needs to calculate the reliability of its neighbors to select the optimal forwarder, thus causing additional delay. Compared with ExOR and RPRDC, in the proposed scheme, the path for a source node to forward its sensing data is calculated in advance, so it has a shorter delay in each hop of data forwarding, which is the main reason that it can achieve almost five times and four times improvement in PDR over ExOR and RPRDC, respectively. In addition, our proposed routing protocol can change the current path adaptively when it is failed, thus blocks the decline of the PDR.

#### 5.2.2. Energy Consumption 

In this section, two scenarios are designed to evaluate the performance of EC in network. In the first scenario, we evaluate the EC for a source node to forward its sensing data to the mobile sink successfully. In the second scenario, we evaluate the total EC in network varies by time.

**Figure 12:**
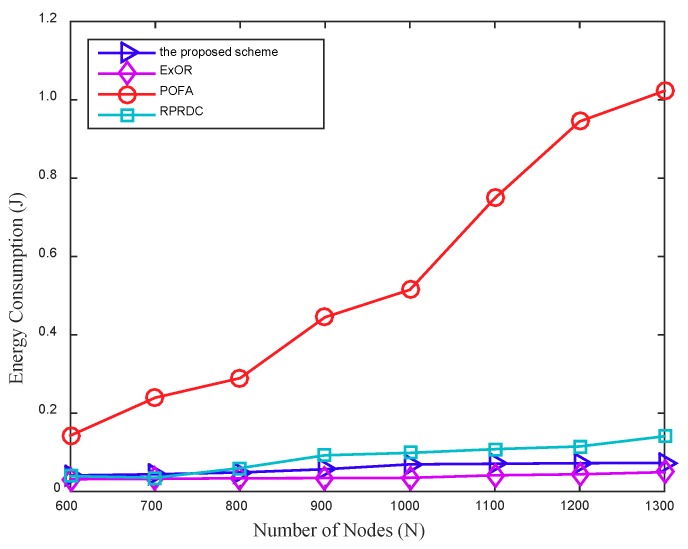
The Energy Consumption (EC) varies by the network density, in addition, $R_{l}$ = 110 m.

Figure 12 shows the EC for a source node to forward its sensing data to the mobile sink varies by the network density. In this work, we select the source node whose distance to the mobile sink is half of $R_{l}$ to conduct this experiment and the EC in this work only reflects the energy consumed by the forwarding process when the source node sends its sensing data to the sink successfully.

**Figure 13:**
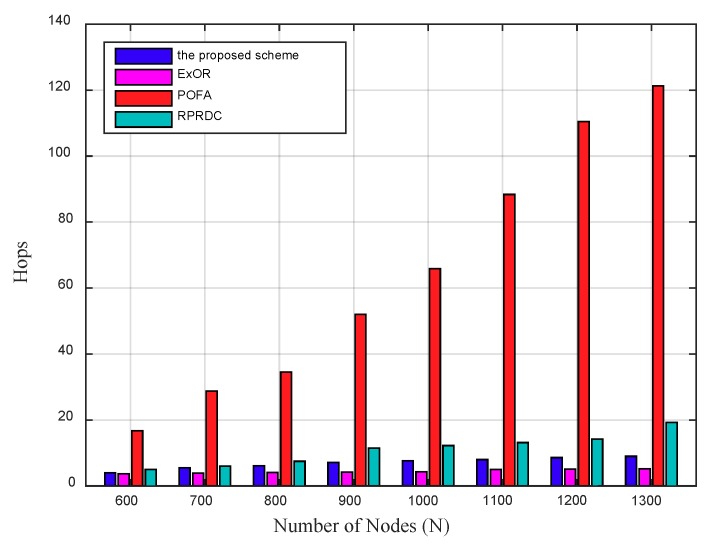
The Hops varies by the network density, in addition, $R_{l}$ = 110 m.

Lots of tests show that the EC increases as the network density expands and it increases more obviously in POFA than that in the proposed scheme, ExOR and RPRDC. The main reason is that the increased number of nodes increases the hops a source node takes in date delivering, as shown in Figure 13, which further increases the EC.

The EC in the proposed scheme is slightly higher than that in ExOR, and they are much lower than that in POFA. The reason is that, in POFA, its data forwarding strategy increases its PDR by selecting multiple forwarders but at the expense of energy, as network density expands, the improved network connectivity increases the success rate of each hop of data forwarding, which further increases hops and the times of reliability calculation it takes, so that the EC in it is always high. In the proposed scheme, the neighbor of a node increases as the network density increases, when a node on the optimal path adjusts the path due to the next hop node is in sleeping status, it needs to select an optimal neighbor that can maximized the PC of the path from it to the sink, this process increases the times of calculation by using the PCs of all neighbors, which further increases the EC.

**Figure 14:**
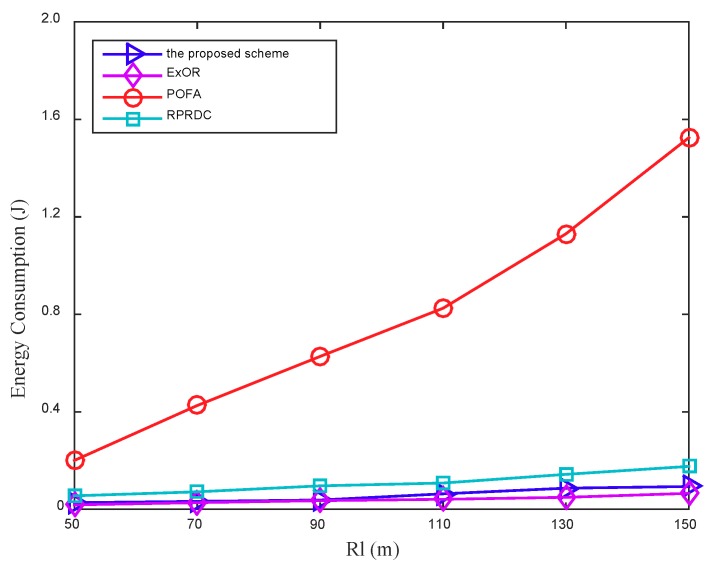
The EC varies by the long radio range $R_{l}$ of the mobile sink, in addition, the number of nodes (N) is 1100.

**Figure 15:**
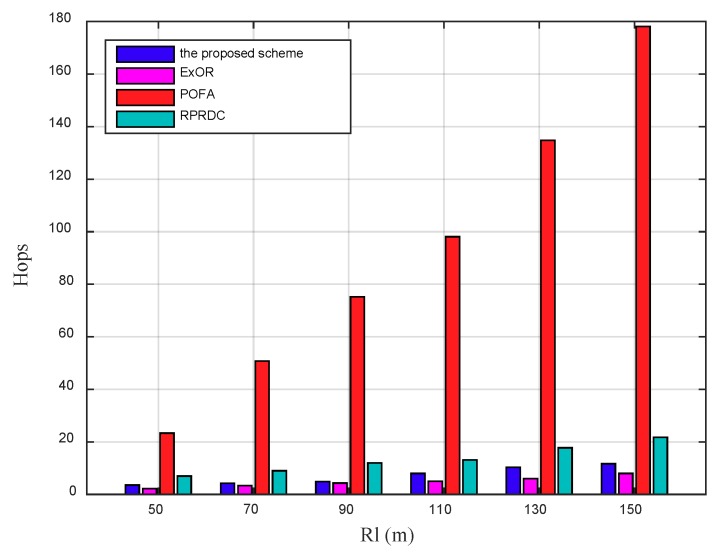
The Hops varies by the long radio range $R_{l}$ of the mobile sink, in addition, the number of nodes (N) is 1100.

Figure 14 shows the EC for a source node to forward its sensing data to the mobile sink grows with the long radio range $R_{l}$ of the mobile sink increases. The node whose distance to the mobile sink is half of $R_{l}$ is selected as the source node. With the increasing of $R_{l}$, the distance from the source node to the mobile sink increases, so the hops it takes in data forwarding also increases, as shown in Figure 15, which further increases the EC.

The EC in the proposed scheme is slightly higher than that in ExOR, but they are much lower than that in POFA. In POFA, each node selects multiple forwarders to achieve the target reliability until one of them connected to the mobile sink, the number of forwarders increases greatly as $R_{l}$ expands, so the increased data delivering and reliability calculation increases its EC. In the proposed scheme, with the expansion of $R_{l}$, the network density keeps unchanged but the distance from the source node to the mobile sink increases, the success rate of data delivering decreases, which further increases the times of calculating the PC to adjust the data forwarding path. This path adjustment scheme can improve its PDR but increase its EC.

**Figure 16:**
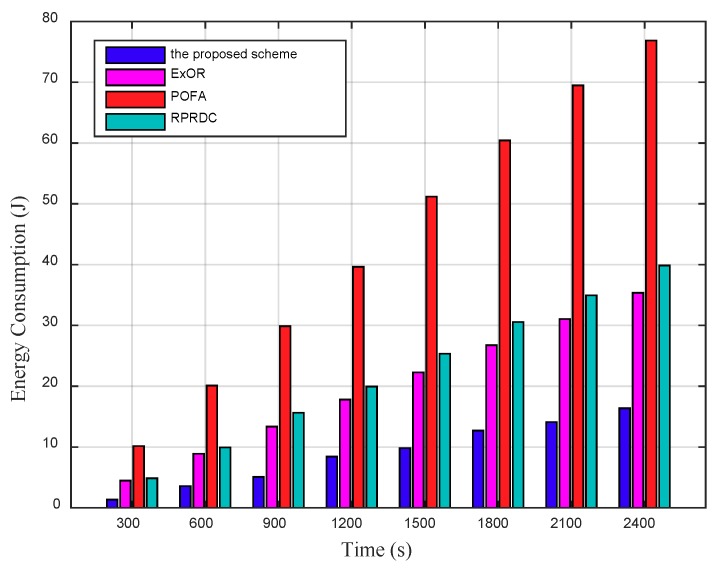
The total EC varies by time in network, in addition, $R_{l}$ = 110 m, the number of nodes (N) is 1100.

Figure 16 shows that the total EC increases by time, in which the mobile sink launches multiple data collection tasks anywhere in network randomly, the proposed scheme consumes 55%, 80% and 61% less energy than that in ExOR, POFA and RPRDC. Here, the energy consumption of nodes is calculated regardless of whether source nodes send their sensing data to the sink successfully or not.

In the proposed scheme, the total EC is mainly consist of the energy consumption caused by the probe message forwarding and the sensing data delivering. By employing the EOH metric, the proposed energy efficient probe message forwarding mechanism can decrease the energy consumption for forwarding the probe message. By analyzing these messages, the mobile sink could construct the spanning tree and broadcast it, so the source node could obtain its optimal path with the max PC to the sink before sending its sensing data. Therefore, during data delivering, a node can save the energy consumed by selecting the forwarders.

#### 5.2.3. Network Lifetime 

In this section, the mobile sink can launch multiple data collection tasks anywhere in network randomly, and we conduct simulations to evaluate the performance of NL according to the number of death nodes, of which the NL is defined as the lifetime for 25 percent nodes that exhaust their energy in network.

In Reference [36], the authors indicated that the death of 25 percent nodes in network could block the WSNs operation greatly, in terms of both the imminent topological disruptions and the decrease of the sensor field’s coverage, which could further hinder the data delivering heavily.

**Figure 17:**
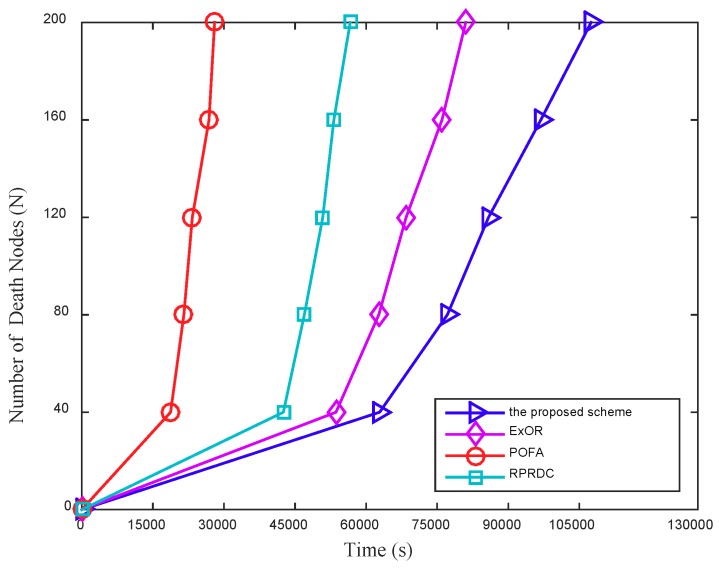
The number of death nodes varies with time, in addition, $R_{l}$ = 90 m, the number of nodes (N) is 800.

Figure 17 shows the number of death nodes increases by time. In this work, the mobile sink moves randomly in network and launches a data collection task with a period set to 600 s. When the task is completed, the sink could move to other locations in network randomly to continue another data collection.

In POFA, the node dies faster than that in the proposed scheme, ExOR and RPRDC. The main reason is that it always seeks to improve the reliability of data transmission in each hop, but at the expense of energy consumption, which speeds up the death of nodes. In addition, the time of the death of 200 nodes (25 percent nodes in network) in the proposed scheme is longer than that in ExOR, POFA and RPRDC. The reason is that, despite the nodes on the optimal paths may take more data forwarding tasks, which can reduce their lifetime and furthermore decreases the network connectivity. However, by calculating the optimal path for nodes in advance to forward the data, and employing the flexible routing protocol that could change the data forwarding path adaptively in the low connectivity network, the proposed scheme consumes less energy of nodes, which is also the reason that as the number of the data collection tasks launched by the mobile sink increases, the time for the death of the same number of nodes in the proposed scheme is getting longer than that in ExOR, POFA and RPRDC.

## 6. Discussion

**Figure 18:**
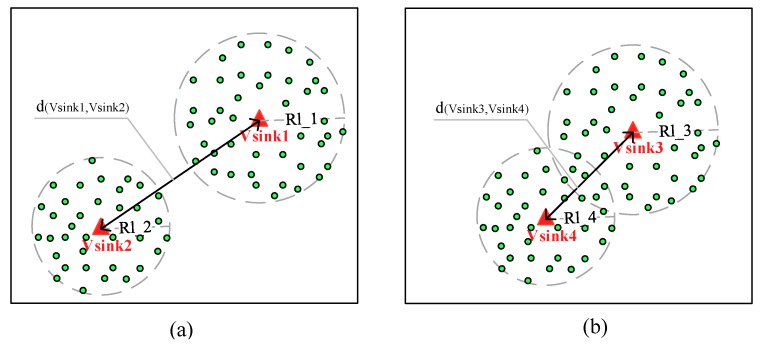
The illustration of two cases when two mobile sinks collect data in network simultaneously, (**a**) the data collection scopes of the mobile sinks are non-overlapped, (**b**) the data collection scopes of mobile sinks are overlapped.

The proposed data collection scheme is also applicable to the scenario where multiple mobile sinks employed in network simultaneously, by considering the distance between the mobile sinks, a feasible scheme is proposed. To show how it works in detail, two mobile sinks are taken as an example, as shown in Figure 18, in which $R_{l}\_1$, $R_{l}\_2$, $R_{l}\_3$ and $R_{l}\_4$ are the long radio ranges of $v_{\sin k1}$, $v_{\sin k2}$, $v_{\sin k3}$ and $v_{\sin k4}$. The mobile sinks move in network randomly, and according to the distance between them, there will be two data collection cases, as shown in Figure 18a,b, respectively.

In Figure 18a, the distance between the two mobile sinks satisfies:

(20) $$d(v_{\sin k1},v_{\sin k2})\geq R_{l}\_1+R_{l}\_2.$$

In this scenario, the mobile sinks could use the proposed data collection scheme to perform their own data collection tasks directly.

In Figure 18b, the distance between the two mobile sinks satisfies:

(21) $$d(v_{\sin k3},v_{\sin k4})<R_{l}\_3+R_{l}\_4.$$

In this case, the data collection scope of the two mobile sinks are overlapped. To clarify the data delivering destination for nodes within the overlapping area, when a node receives a tag message for the first time, it takes this SID of a mobile sink included in the message as its data forwarding destination until the data collection task is completed, and during this process, it neglects other mobile sinks’ data collection task even if it receives the tag messages they broadcast.

This scheme increases the flexibility of data collection, and by using multiple mobile sinks, it can collect data from multiple network regions simultaneously, which is applicable in prospect applications.

## 7. Conclusions

In this paper, we propose a novel data collection scheme based on opportunistic node connections in network with a mobile sink, which can be used in smart industrial fields. In the scheme, the mobile sink can launch a data collection task anywhere in network at any time by broadcasting a tag message, a node that receives this message can forward its probe message that includes its source information to the mobile sink by using the energy efficient probe message forwarding mechanism to minimize the overall energy consumption. On receiving these messages, the mobile sink first adopts the random graph theory to constructs an opportunistic connection random graph, then calculates the optimal path from itself to each node in this graph, hence a spanning tree is generated where the mobile sink is the root node, and it finally broadcasts this spanning tree to all nodes under its long radio range so that they can obtain the optimal paths from themselves to the mobile sink. Accordingly, a routing protocol which adapts to different nodes operating status is proposed to improve the reliability of data transmission. The simulation results show that the proposed scheme works better concerning the packet delivery ratio, energy consumption and network lifetime.

The possible future work are, first, studying the synergy between mobile sinks, and exploring the impact of the comprehensive factors such as the number of mobile sinks and the their long radio ranges on data collection efficiency. Second, studying the energy efficient cross-layer protocol design [37], so that it could be used more widely. Third, inspired by work in Reference [38], of which the authors proposed a fog structure composed of multiple mobile sinks that act as fog nodes to bridge the gap between WSNs and the Cloud, it can consider using mobile sinks to upload data to the cloud and use their powerful computing and storage capability to improve the efficiency of constructing the OCRG and calculate the optimal paths.

## Figures and Tables

**Table 1:** The symbols and parameters.

Symbols	Description
*r*	Radio range for sensor nodes
$R_{l}$	Long radio range for the mobile sink
$R_{s}$	Short radio range for the mobile sink
$v_{s}$	Source node
$v_{\sin k}$	Mobile sink
N	Number of nodes in a data collection scope
∅	Empty set

**Table 2:** The format of the probe message.

**Message Source**	**Source Information**	**Sink ID**	**Forwarders ID**	**EOH**

**Table 3:** The Path Connectivity (PC) of the path from $v_{\sin k}$ to each node.

Step	$\mathrm{PC}[v_{1}]$	$\mathrm{PC}[v_{2}]$	$\mathrm{PC}[v_{3}]$	$\mathrm{PC}[v_{4}]$	$\mathrm{PC}[v_{5}]$	$\mathrm{PC}[v_{6}]$
Initialization	0.7	0.6	0.4	0	0	0
1	**0.7**	0.6	0.4	0	0	0.441
2	0.7	**0.6**	0.4	0.228	0.228	0.441
3	0.7	0.6	0.4	0.228	0.318	**0.441**
4	0.7	0.6	**0.4**	0.228	0.318	0.441
5	0.7	0.6	0.4	0.228	**0.318**	0.441
6	0.7	0.6	0.4	**0.228**	0.318	0.441

The value with bold format is the max value of PC in set U.

**Table 4:** Simulation parameters.

Parameters	Values
Network Size	400 × 400 m^2^
Number of the Mobile Sink	1
Mobility Pattern	Randomly
Duration for a Data Collection Period	600 s
Communication Range for Sensor Nodes	20 m
Data Packet Size	1024 bits
Probe Message Size	120 bits
Target Reliability in Probabilistic and Opportunistic Flooding Algorithm (POFA)	0.6
Initial Energy of Nodes	2 J
Buffer Size of Nodes	1024 bits
$E_{elec}$	20 × 10^−7^ J/bit
$\varepsilon_{amp}$	10 × 10^−9^ J/bit/m^2^
